# Hyperspectral inversion model of ginkgo leaf yield prediction based on machine learning

**DOI:** 10.3389/fpls.2025.1698830

**Published:** 2025-11-28

**Authors:** Zheng Zuo, Maocheng Zhao, Liang Qi, Bin Wu, Hongyan Zou, Weijun Xie, Qiaolin Ye, Chi Zhou, Kai Zhang

**Affiliations:** 1 College of Mechanical and Electronic Engineering, Nanjing Forestry University, Nanjing, China; 2 Jinpu Research Institute, Nanjing Forestry University, Nanjing, China; 3 College of Information Science and Technology, Nanjing Forestry University, Nanjing, China

**Keywords:** hyperspectral imaging, analysis, spectral index, ginkgo biloba leaves, non-destructive solution

## Abstract

**Introduction:**

The yield of ginkgo biloba leaves serves as a critical indicator for assessing their growth and health status. However, current assessment methods primarily rely on manual harvesting and weighing, which are time-consuming, labor-intensive, inefficient, and costly.

**Methods:**

To address these limitations, this study designed an algorithm-based yield estimation approach: by employing airborne hyperspectral imaging technology at a research base to replace traditional manual measurements, a canopy hyperspectral dataset and Region of Interest Pixel (ROP) sets were constructed. Five preprocessing methods, Multiplicative Scatter Correction (MSC), Standard Normal Variate (SNV), Savitzky-Golay (SG), First Derivative (FD), and Standard Scaling (SS), were employed to develop Partial Least Squares Regression (PLSR) models, identifying the optimal hyperspectral data preprocessing approach. The optimal preprocessing model was subsequently integrated with Particle Swarm Optimization (PSO), Successive Projections Algorithm (SPA), Principal Component Analysis (PCA), Least Absolute Shrinkage and Selection Operator (LASSO), Competitive Adaptive Reweighted Sampling (CARS) and Particle Swarm Attention Mechanism Algorithm (PSAMA) for feature band selection. Traditional spectral vegetation indices were refined through random forest stepwise regression and spectral index correlation analysis, ultimately determining Soil-Adjusted Vegetation Index (SAVI), Modified Soil-Adjusted Vegetation Index (MSAVI), Normalized Difference Red Edge Index (NDRE), Structure Insensitive Pigment Index (SIPI) as the final indices. The selected spectral bands and vegetation indices were then incorporated with PLSR, Random Forest (RF), K-Nearest Neighbors Regression (KNNR), Long Short-Term Memory (LSTM), Support Vector Regression (SVR), Bidirectional LSTM (BiLSTM), and BiLSTM- Grid SearchCV (BiLSTM-GS) machine learning models for yield prediction.

**Results:**

Results demonstrated that the SNV-PLSR model achieved superior performance ( $R_{p}^{2}$ = 0.7831, $RMSE_{P}$ = 0.0325). The optimal SNV- (SAVI - MSAVI - NDRE - SIPI - ROP) - (BiLSTM-GS) model, combining PSAMA-selected feature bands with vegetation index and ROP, yielded outstanding prediction accuracy ( $R_{p}^{2}$ = 0.8795, $RMSE_{P}$= 0.1021).

**Discussion:**

This airborne hyperspectral canopy-based estimation technology provides an accurate, non-destructive solution for monitoring ginkgo leaf yield in field cultivation.

## 1 Introduction

As an economically important medicinal plant, Ginkgo biloba is cultivated worldwide for its leaves, which serve as the primary raw material for pharmaceutical and health products (Liu et al., 2022; Lu et al., 2024). According to modern pharmacological research, Ginkgo biloba has a promoting effect on protecting nerve cells, anti-oxidation and scavenging free radicals at the body (Vitor et al., 2023; Sen et al., 2025). In the growth process of Ginkgo biloba, its yield plays a vital role, which is one of the important factors that determine the growth of Ginkgo biloba (Li et al., 2024). Traditional ginkgo leaf yield test methods, such as scale weighing method, are usually only suitable for small plot experiments, and there are problems such as time-consuming, laborious operation, and destruction of plant growth. To address the above problems, researchers have proposed a new detection method, that is, airborne hyperspectral canopy ginkgo leaf image to predict ginkgo leaf yield. This method not only saves time, effort and protects plant growth, but also is suitable for the detection of large-scale plots.

Research on the detection of plant yield based on hyperspectral technology has gradually increased. Different plant yield estimation models are established mainly through hyperspectral image data preprocessing (Jin et al., 2023), feature band selection, and machine learning model algorithms (Li et al., 2025; Yan et al., 2025). For example, Qin et al. (2025) used the ASD hyperspectral sensor to collect the hyperspectral yield data of apple tree canopy in the whole growth stage of spring and autumn. First, the convolution Savitzky-Golay (SG) was used to preprocess the hyperspectral data. Second, the sensitive bands in hyperspectral data were screened by Genetic Algorithm (GA) and Successive Projections Algorithm (SPA). Finally, the selected bands were combined with machine learning models such as PLSR, RF, and XGBoost to predict the hyperspectral yield data of apple trees collected in spring and autumn. The results showed that the data collected in autumn performed better in prediction performance. The best model combination was SG- (VISSA-CARS) -RF, with *R^2^* of 0.78 and RMSE of 6.03 in the validation set.

Burglewski et al. (2024) used a hyperspectral imager to collect spectral data of corn straw, aiming to estimate the yield of corn as silage. In the study, seven spectral bands including red edge bands and multiple near-infrared bands were used to predict the yield combined with the Support Vector Regression (SVR) model. The results show that the method can achieve more than 90% prediction accuracies (Mao et al., 2024). predicted wheat yield and yield loss under water and nitrogen stress using hyperspectral remote sensing data and Partial Least Squares Regression (PLSR) models. In this study, researchers paired canopy hyperspectral data from the same location, at the same growth stage but under different stress conditions with yield data to form a data combination for verifying the performance of the model. The results showed that compared with the traditional PLSR model, the MRE-PLSR model significantly improved the prediction accuracy, and the Pearson correlation coefficient increased by an average of 14.5%.

Ceriani et al. (2025) integrated hyperspectral and LiDAR space-borne data to estimate forest volume and biomass in mountainous regions. By combining LiDAR-derived metrics (e.g., canopy height) with hyperspectral indices, they used machine learning models like Random Forest (RF) and Support Vector Regression (SVR) to predict aboveground biomass and forest volume. The results showed that the fusion of both datasets improved accuracy, with an *R*^2^ value above 0.85 and a 20% reduction in root mean square error. This highlights the potential of using integrated remote sensing data for large-scale forest assessments. Miettinen et al. (2024) utilized hyperspectral imaging to reveal the spatiotemporal dynamics of chlorophyll and carotenoids in Scots pine under water stress. Among the multiple machine learning models evaluated, the Random Forest Regression (RFR) model demonstrated the best predictive performance, with predictions most closely aligned with measured values and the lowest Root Mean Square Error (RMSE). Tougas et al. (2025)captured the weak spectral features of the early stage of beech tree disease by hyperspectral imaging technology, and systematically evaluated the classification performance of various machine learning models. The results showed that the random forest (RF) model performed best in this task, with a classification accuracy of 85%, which was significantly ahead of other models, confirming the effectiveness and application potential of hyperspectral technology combined with RF model in early diagnosis of forest diseases.

While previous studies have extensively applied hyperspectral remote sensing to crop monitoring, research on ginkgo leaf yield prediction by integrating hyperspectral technology with machine learning remains relatively scarce. Building on existing methodologies, this study employs airborne hyperspectral imaging and electronic weighing for efficient and non-destructive data acquisition of ginkgo leaf spectra and yield. The proposed approach is operationally straightforward, time-efficient, and suitable for field applications. The main innovations of this work are summarized as follows:

1. To achieve the efficient screening of hyperspectral features, an attention mechanism model is integrated into the Particle Swarm Optimization (PSO) algorithm, leading to the construction of the PSAMA optimization algorithm.
2. The integration of optimally selected feature bands, refined spectral vegetation indices, and ginkgo canopy Region of Interest Pixel (ROP) sets can significantly enhance both the accuracy and reliability of yield prediction models.
3. A novel SNV- (SAVI - MSAVI – NDRE - SIPI - ROP) - (BiLSTM-GS) prediction model is proposed, which achieves excellent detection performance through innovative architecture design and optimization training.

## 2 Materials and methods

**Figure 1:**
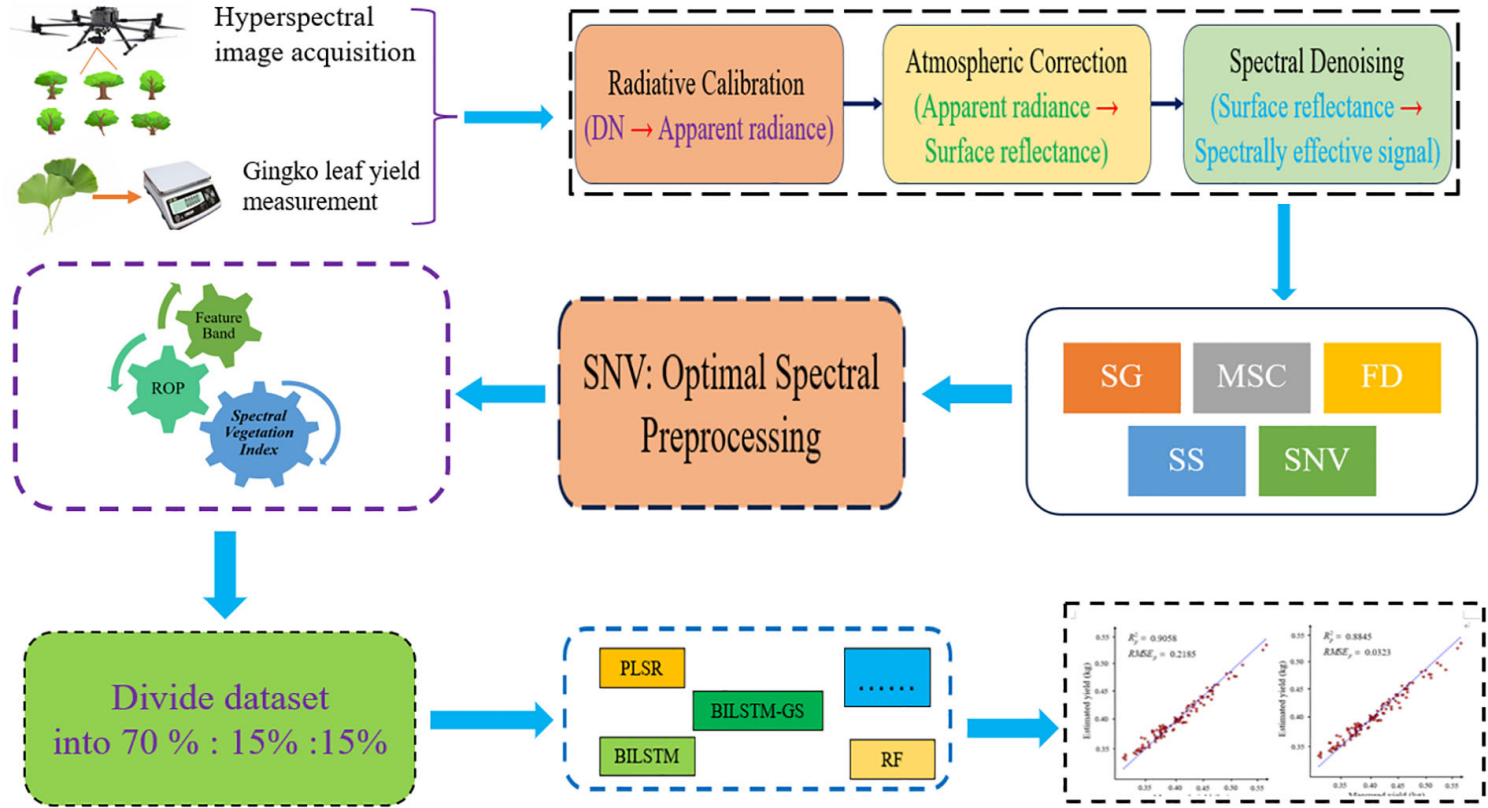
The overall flow chart of the experiment. The schematic diagram illustrates the relationship between the obtained hyperspectral canopy ginkgo leaf reflectance, canopy ginkgo leaf yield and the prediction model.

The overall workflow of this study is illustrated in **Figure 1**, which outlines the complete process of hyperspectral data acquisition, preprocessing, feature extraction, and model construction for Ginkgo biloba leaf yield estimation. This schematic provides an overview of how canopy hyperspectral reflectance, ginkgo leaf yield, and the prediction model are interrelated.

### 2.1 Experimental site and period

**Figure 2:**
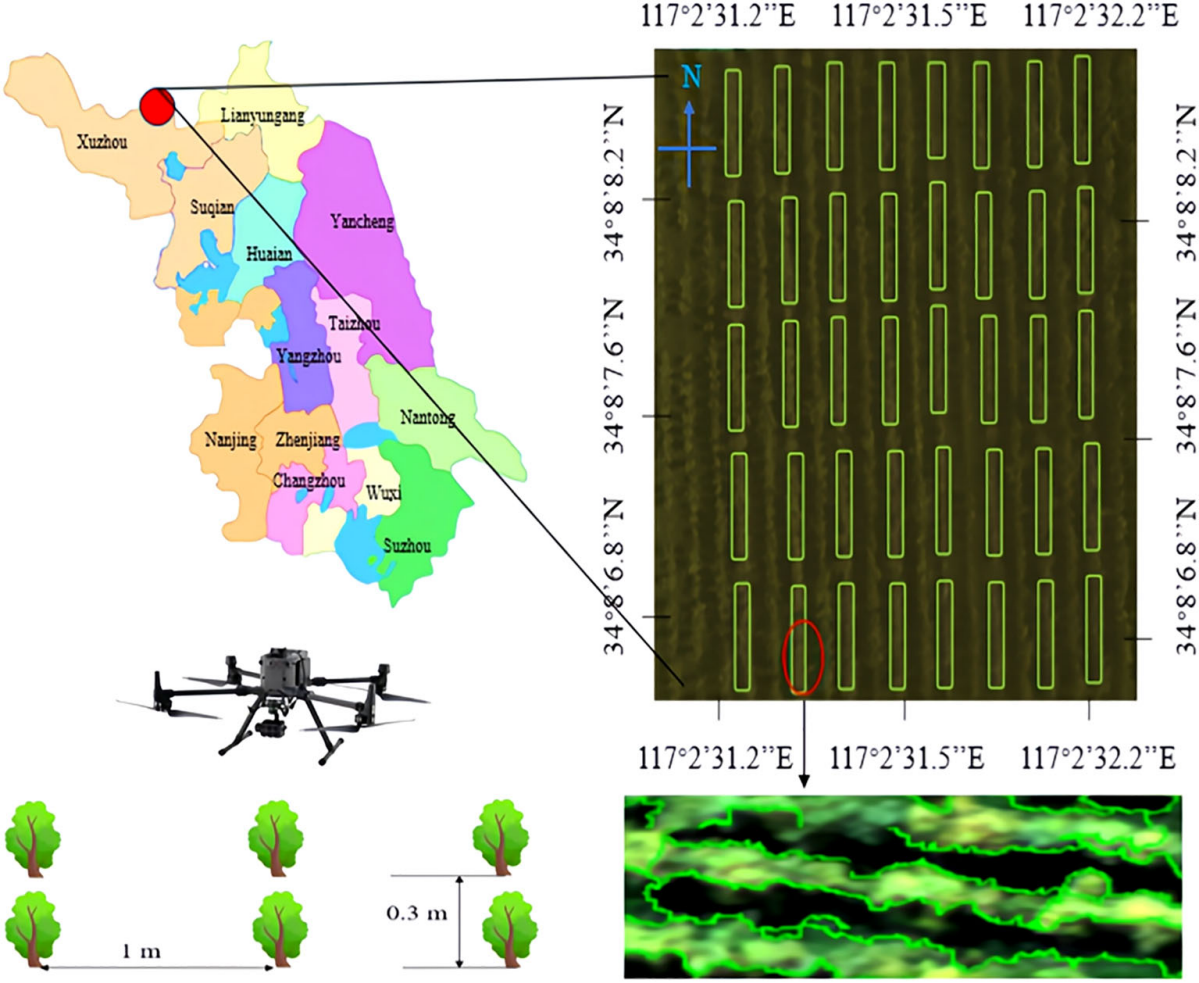
Ginkgo biloba scientific research base and ginkgo leaf collection method.

Hyperspectral remote sensing images of Ginkgo biloba canopy were acquired for yield acquisition. The experimental site was Xuzhou Changrong Agricultural Development Co., Ltd. (34°8 ‘ 42’N, 117°2 ‘45’E). A total of 10,000 Ginkgo biloba trees were planted in the scientific research base, covering a plot size of 0.8 × 4 m^2^. The experimental involved three-year-old ginkgo trees cultivated at the scientific research base. The trees were spaced 1 m between rows and 0.3 m between plants. **Figure 2** shows the geographical location of the experimental site and the contour characteristics of the ginkgo tree canopy.

### 2.2 Hyperspectral remote sensing image acquisition of canopy ginkgo leaves

In this study, ginkgo leaves in the experimental site were selected as the research object, and the remote sensing data of ginkgo leaf canopy were obtained by using an airborne hyperspectral imaging system (GaiaSky-mini3-VN, Double-profit Hepu Technology Co., Ltd., China). The hyperspectral imaging system used in this study has a spectral response range of 400 ~ 1000 nm, equipped with 224 spectral channels and 1024 spatial channels, which can achieve a spatial resolution of 1024 × 1003 pixels and a spectral resolution of 5 nm. The lens carried by the imaging system has an optical resolution of 16 nm, and the overall power consumption of the system is 45 W. The UAV was used as the data acquisition platform, and the flight height was fixed at 50 m (DJI M350, DJI Innovation Technology Co., Ltd., China). During data acquisition, the drone maintains a hover for 10 s, and continuous image acquisition is performed through the built-in push-scanning mode of the hyperspectral imager to ensure spatial continuity and spectral consistency of the data. The hyperspectral canopy images of ginkgo leaves were acquired on July 19,2024 and September 15,2024. The weather was sunny, with temperatures of 30 °C and 24 °C, relative humidities of 75% and 68%, and wind speeds of 8.6 km/h and 6.4 km/h, respectively.

### 2.3 Field measurement of ginkgo leaf yield

From July 19 to 26, 2024 and from September 15 to 21, 2024, the laboratory team successfully completed the acquisition of hyperspectral images of ginkgo tree canopy leaves at the research base. Upon concluding the image collection, the team grouped the leaves from every twenty ginkgo trees into one sample unit, obtaining a total of 200 sample datasets. Subsequently, an electronic balance (manufactured by Kunshan Toptech Electronic Co., Ltd., featuring functions such as counting, pricing, and weighing) was used to precisely weigh the leaves from all 200 samples, and the corresponding weight data was systematically recorded.

### 2.4 Hyperspectral image correction

Hyperspectral imagery, comprising hundreds of contiguous spectral bands, is inherently susceptible to degradation from environmental conditions (Yue et al., 2024), atmospheric effects (Andrei et al., 2023), and sensor-related errors (Settembre et al., 2025). To mitigate these impacts, a series of radiometric and atmospheric corrections are essential (Black et al., 2024). For instance, a fundamental step entails converting raw digital numbers (DN) to surface reflectance using methods such as the Empirical Line Method (ELM), which can be represented by the linear transformation in Equation 1:

(1) ρ=G×DN+O

Where ρ is the derived surface reflectance, G is the gain coefficient, DN is the raw digital number from the sensor, and O is the offset coefficient.

These correction procedures significantly enhance the data’s radiometric accuracy and spectral fidelity, thereby establishing a reliable foundation for subsequent quantitative analysis and image processing (Gila et al., 2024).

### 2.5 ROI extraction of hyperspectral image

The ROI (Region of Interest) tool in ENVI 5.3 was used to extract the target regions from the hyperspectral canopy images of Ginkgo biloba, thereby obtaining the ROP sets. By using the average value method to aggregate the spectral data of the target area, a high-quality data set representing the spectral characteristics of ginkgo canopy was constructed. This method effectively eliminates the interference of background noise and abnormal pixels, significantly improves the reliability and representativeness of the data, and lays a solid data foundation for subsequent spectral feature analysis and model construction (Wang X, et al., 2024).

### 2.6 Hyperspectral data preprocessing

Hyperspectral images are often influenced by environmental and instrumental factors such as temperature, humidity, light intensity, and illumination angle, which can introduce noise into the data. To improve data quality, a series of preprocessing steps were applied, including Multiplicative Scatter Correction (MSC) (Xu et al., 2024), Savitzky–Golay smoothing (SG) (Bing et al., 2018), Standard Normal Variate (SNV) (Jong et al., 2024), First Derivative (FD) (Xunlan et al., 2023), and Standard Scaling (SS) (Faehn et al., 2024).

### 2.7 Construction method of spectral vegetation index

Among the alternative spectral indices, those with a high correlation to ginkgo leaves and a weak intercorrelation were selected. Based on the spectral indices used in relevant references, seven commonly used spectral indices are chosen as alternative indices, and their respective calculation formulas are shown in **Table 1**. The Normalized Difference Vegetation Index (NDVI), obtained by the ratio of the near-infrared to the red band, reflects the vegetation coverage and Leaf Area Index (LAI), and is often used to assess the health status and biomass of vegetation (Wang et al., 2024). The Red Edge Chlorophyll Index (ReCI), taking advantage of the sensitivity of the red edge band to chlorophyll content, directly reflects the chlorophyll concentration in leaves (Xueyu et al., 2021). The NDRE, calculated as the ratio of the near-infrared to the red edge band, is more sensitive to changes in chlorophyll content, especially in the later stages of vegetation growth. The Green Normalized Difference Vegetation Index (GNDVI), which replaces the red band with the green band, is more sensitive to changes in chlorophyll content (Ge et al., 2019). The SAVI, by introducing a soil adjustment factor, reduces the influence of the soil background on the vegetation index and more accurately reflects the vegetation coverage (Tooley et al., 2024). The SIPI is insensitive to changes in leaf structure and mainly reflects the ratio of carotenoids to chlorophyll (Srivastava et al., 2020). The MSAVI further optimizes the impact of the soil background and is applicable to areas with low vegetation coverage (Shezhou et al., 2015). These seven spectral indices have a high correlation in the prediction of ginkgo leaf yield and a relatively weak correlation with each other. By covering key factors such as leaf area and chlorophyll content, they can effectively evaluate the health status, photosynthetic efficiency, and biomass of ginkgo leaves, providing a scientific basis for yield prediction.

**Table 1:** Operation formula of spectral vegetation index.

Spectral Index	Computing Formula
NDVI	(NIR_801_ – RED_682_)/(NIR_801_ + RED_682_)
ReCI	(NIR_801_/RED_701_) -1
NDRE	(NIR_801_ – RED EDGE_720_)/(NIR_801_ + RED EDGE_720_)
GNDVI	(NIR_801_ – GREEN_561_)/(NIR_801_ + GREEN_561_)
SAVI	((NIR_820_ – RED_680_)/(NIR_820_ + RED_680_ + L)) * (1 + L)
SIPI	(NIR_820_ – BLUE_462_)/(NIR_820_ – RED_712_)
MSAVI	0.5[(2NIR_801_ + 1 – SQRT((2NIR_801_ + 1)^2^ – 8 (NIR_801_– RED_682_))]

Where because ginkgo biloba belongs to the low green vegetation area, L = 1.

### 2.8 Characteristic band screening

To effectively reduce the complexity and redundancy of hyperspectral data, this study employs feature band selection methods to enhance data processing efficiency and improve the accuracy and reliability of yield prediction models by identifying key bands most relevant to ginkgo leaf yield. The hyperspectral dataset was first divided into training, validation, and test sets in a 70%:15%:15% ratio to ensure the independence of model evaluation. Subsequently, feature band selection was performed using Particle Swarm Optimization (PSO) (Wei et al., 2024), Successive Projections Algorithm (SPA) (Souza et al., 2025), Principal Component Analysis (PCA) (Matinfar et al., 2025), Least Absolute Shrinkage and Selection Operator (LASSO) ( (Stevens et al., 2025), Competitive Adaptive Reweighted Sampling (CARS) (Shi et al., 2024), Spectral Index (SI) (Wang et al., 2024), and the Particle Swarm Attention Mechanism Algorithm (PSAMA). The working principles of all algorithms will be systematically elaborated below, with particular emphasis on PSO and its improved version, PSAMA.

The core idea of PSO is to explore and find the optimal solution by simulating a group of particles in the search space. Each particle represents a possible solution. Through ‘flying’ in the search space, the particle swarm gradually adjusts its position by using its own historical experience and group collaboration information, and finally converges to the global optimal solution (Ren et al., 2024), as shown in Equations 2 and 3.

(2) $$v_{i}^{d}=w*v_{i}^{d-1}+c_{1}*r_{1}*(pbest_{i}^{d}-x_{i}^{d})+c_{2}*r_{2}*(gbest_{i}^{d}-x_{i}^{d})$$

(3) $$x_{i}^{d+1}=x_{i}^{d}+v_{i}^{d}$$

Where $c_{1}$ is individual learning factor, $c_{2}\;$ social learning factor, w velocity inertia weight, $v_{i}^{d}$ the speed of the d th iteration of the i th particle, $x_{i}^{d}$ the position of the d th iteration of the i th particle, $pbest_{i}^{d}\;$the i th particle iterates to the best position of the d th iteration, $\text{g}best_{i}^{d}$ all particles iterate to the best position by d times.

**Figure 3:**
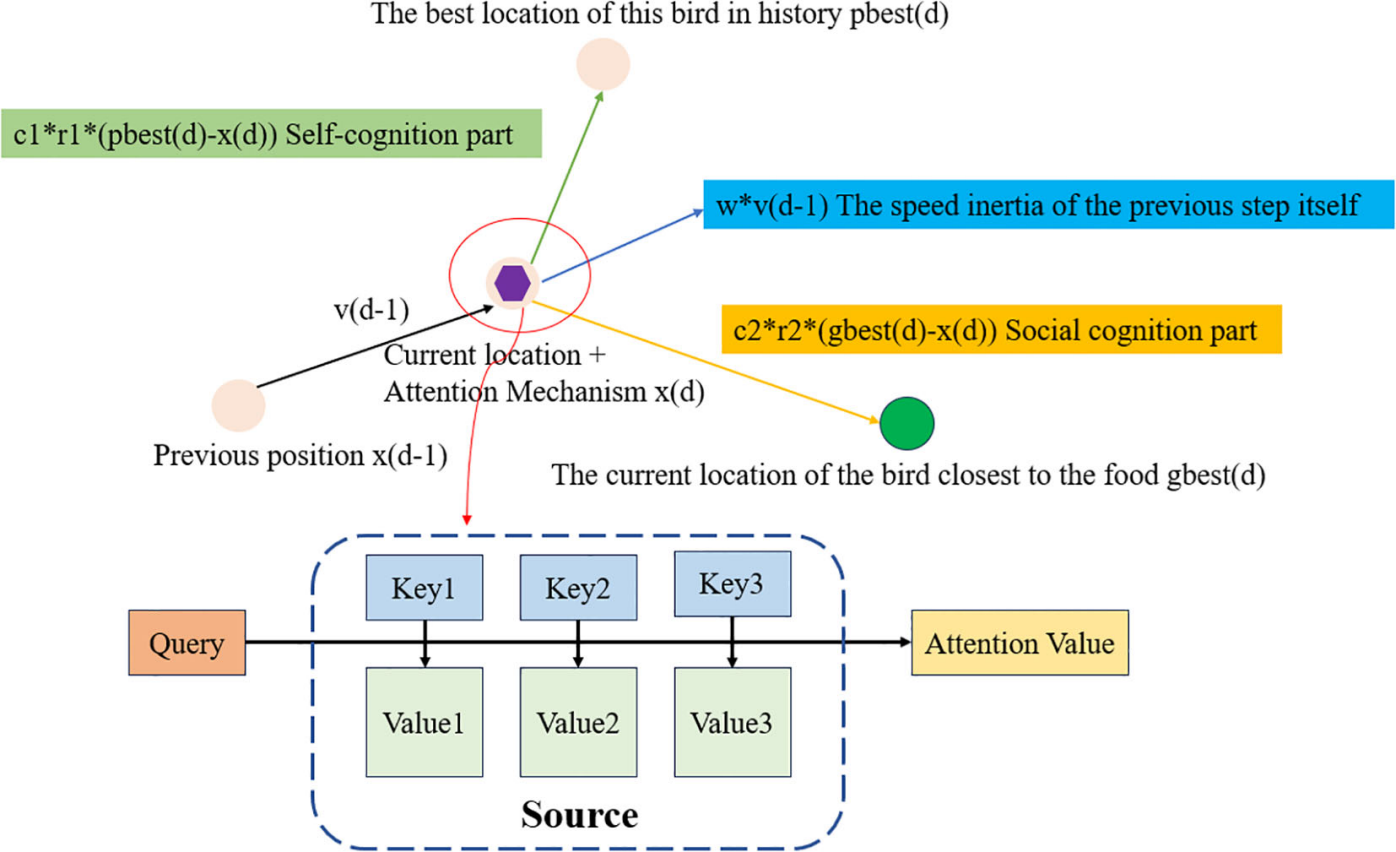
PSAMA working principle diagram. Where Source b is composed of a series of <Key, Value> key-value pairs, Query is a given Target element, Key is the Key value of the element in Source, Value is the value of the element in Source, Similarity or correlation between Query and key, the weight coefficient is Similarity (Query, Keyi), Attention Value is a weighted sum of Value values.

PSAMA mainly introduces the Attention Mechanism into the PSO algorithm, which dynamically adjusts the attention degree of information by adjusting the position of particles. The core idea is to calculate the weight coefficient of each key ‘s corresponding value (Value) according to the correlation between Query and Key, and then obtain the final output by weighted summation, that is, the final Attention values. In essence, the Attention mechanism is the weighted summation of the Value values of the elements in the Source, and Query and Key are used to calculate the weight coefficient of the corresponding value (Lang et al., 2024). Its working principle is shown in **Figure 3** and Equation 4.

According to the principle of attention mechanism, the calculation formula is as follows:

(4) $$Attention(Query,Source)=\sum_{i=1}^{L_{x}}Similarity(Query,Key_{i})*Value_{i}$$

Where $L_{x}=∥Source∥$ denotes the length of Source.

### 2.9 Machine learning and model validation

The optimal model was selected from seven machine learning regression methods, all employing Grid Search Cross-Validation (GridSearchCV) for hyperparameter optimization. The methods and their respective tuned hyperparameters include: PLSR (Sun et al., 2024) with n_components; RF (Sun et al., 2024) with n_estimators; KNNR (Song et al., 2025) with n_neighbors; LSTM (Satyabrata et al., 2024) with learning rate; SVR (Tian et al., 2025) with regularization parameter C; BiLSTM (Sun X, et al., 2024) with learning rate; and BiLSTM-GS, which simultaneously optimizes both learning_rate and hidden_layer sizes.

**Figure 4:**
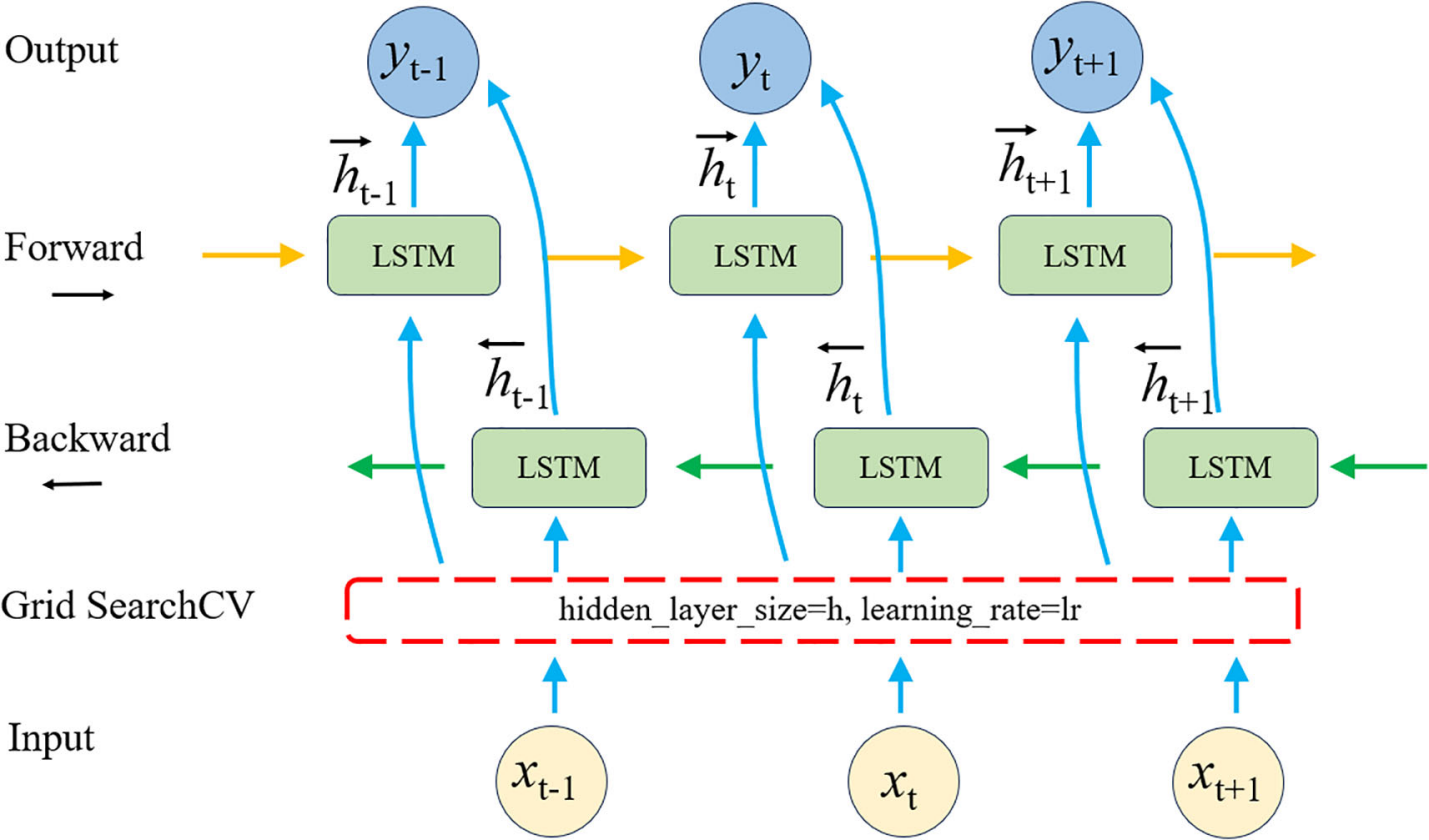
(BiLSTM-GS) working principle diagram.

The BiLSTM-GS model integrates the BiLSTM architecture with the GridSearchCV algorithm (Liang et al., 2024). While the standard BiLSTM model only tunes the learning rate, BiLSTM-GS introduces an additional hyperparameter—hidden layer size—to achieve more comprehensive optimization. As an enhanced LSTM variant, BiLSTM processes sequences bidirectionally (forward and backward), enabling more effective capture of contextual dependencies (Zhang et al., 2025). GridSearchCV automates hyperparameter selection through systematic cross-validation, significantly improving model performance. The architecture of the BiLSTM-GS model is illustrated in **Figure 4**.

Building upon the bidirectional processing capability of BiLSTM, this study leverages its strength in modeling the continuous spectral-temporal sequences of ginkgo leaves. The network architecture enables integrated learning of both forward and backward dependencies within the spectral data, effectively capturing the cumulative physiological changes during leaf growth. This approach significantly enhances the characterization of long-term developmental trends, thereby improving the robustness of yield prediction.

During model configuration, particular attention was given to the interaction between hidden layer dimensionality and learning rate (Nikzad et al., 2025). The hidden layer structure governs the model’s capacity to represent complex spectral patterns, while the learning rate regulates the convergence behavior during training. Proper coordination between these two parameters ensures stable gradient propagation while preventing either underfitting due to insufficient model expressivity or overfitting caused by excessive parameterization, ultimately leading to optimized generalization performance (Wan et al., 2024).

The determination coefficient *R^2^* and the root mean square error RMSE are used as the evaluation indexes of the regression model. The larger the *R^2^* of the model, the smaller the RMSE, and the better the training effect of the model (Long et al., 2024), where $R_{c}^{2}$, $RMSE_{c}$ is the results of the training set, and $R_{p}^{2}$, $RMSE_{p}$ is the results of the calibration set. The calculation formulas are shown in Equations 5 and 6.

(5) $$R^{2}=1-\frac{\sum_{i=1}^{N}{\left(y_{i}-\hat{y_{i}}\right)}^{2}}{\sum_{i=1}^{N}{\left(y_{i}-\bar{y}\right)}^{2}}$$

(6) $$RMSE=\sqrt{\frac{1}{N}{\sum_{i=1}^{N}\left(y_{i}-\hat{y_{i}}\right)}^{2}}$$

Where *N* is the number of samples, $y_{i}\;$ is the actual value of the i th sample, ${\hat{y}}_{i}$ is the actual value of the ith sample, and $\bar{y}$ is the actual mean value of all samples.

### 2.10 Experimental platform and computing environment

All experiments were conducted on a workstation equipped with an Intel Core i7-12700H CPU and 32 GB of RAM. The entire training and hyperparameter tuning process for all models was performed on this CPU platform without GPU acceleration. The software environment utilized PyCharm 2023.2.4 (Community Edition) with Python 3.9, incorporating key libraries such as PyTorch (1.8), scikit-learn (1.2.2), and TensorFlow (2.10.0). The complete workflow required approximately 10 minutes on the specified hardware, demonstrating the computational efficiency of the proposed methodology on standard computing resources.

## 3 Results and discussion

### 3.1 Spectral pretreatment

**Figure 5:**
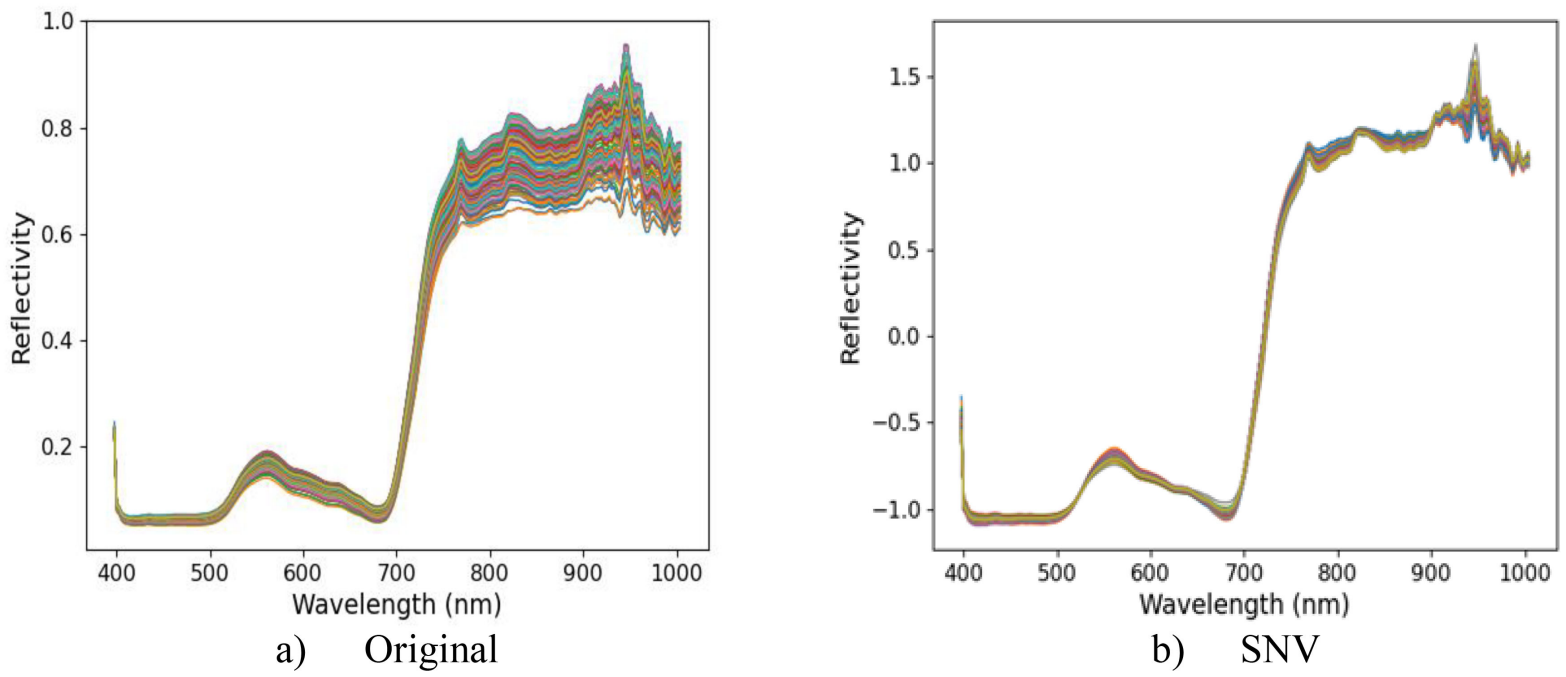
**(a)** shows the original hyperspectral reflectance of ginkgo canopy leaves, and **(b)** shows the hyperspectral reflectance after optimal pretreatment.

Five spectral data preprocessing methods (MSC, SNV, SG, FD, and SS) and PLSR were used to establish regression models for leaf yield of different Ginkgo trees. The prediction results of each model are shown in **Table 2**. The $R_{c}^{2}$ and $R_{P}^{2}$ of different models were higher than the original spectrum, which can be used to detect the yield of ginkgo leaves. Among these models, the SNV model exhibited the highest accuracy with $R_{P}^{2}\;$ = 0.7831 and $RMSE_{p}$ = 0.0325. Therefore, the SNV pretreatment model was used as the basis for subsequent data processing. The hyperspectral original reflectance and the best pretreatment reflectance are shown in **Figure 5**. The weak absorption peak near 820 nm was related to the stretching vibration of the N-H triplet state (Chitra et al., 2022). A smaller absorption peak appears near 930 nm, mainly due to the dual-frequency absorption of O-H (Chitra et al., 2022). The peaks and troughs near 560 nm and 690 nm were caused by chlorophyll and carotenoids at ginkgo leaves (Marchese et al., 2024). These spectral characteristics further confirmed the intrinsic relationship between the chemical composition of Ginkgo biloba leaves and their spectral information, providing strong support for further understanding the spectral characteristics of Ginkgo biloba leaves.

**Table 2:** Comparative analysis of different pretreatment methods and the original spectral PLSR model.

Different regression models	Pretreatment method	Calibration set		Prediction set	
		$R_{C}^{2}$	$RMSE_{c}$	$R_{P}^{2}$	$RMSE_{p}$
PLSR	Original	0.9217	0.0312	0.7658	0.0218
	MSC	0.9345	0.0217	0.7532	0.0445
	SNV	0.9624	0.0232	0.7831	0.0325
	SG	0.9021	0.0147	0.6957	0.0544
	FD	0.8912	0.0322	0.6621	0.0467
	SS	0.8845	0.0455	0.6327	0.0171

### 3.2 Spectral vegetation index selection

**Figure 6:**
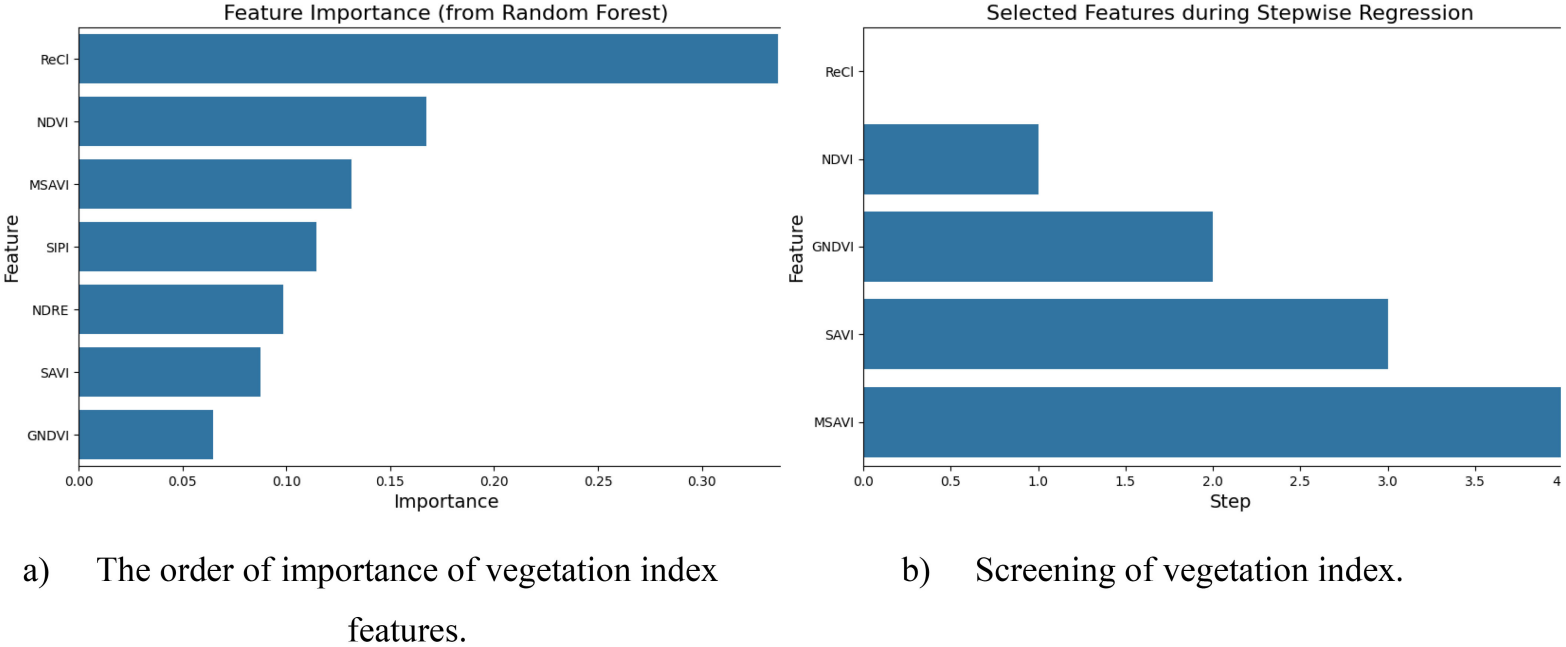
**(a)** shows the vegetation indices screened based on importance ranking, and **(b)** shows the reasonable vegetation indices selected by the stepwise regression method.

In this study, the random forest stepwise regression method was used to select the vegetation index with less redundant information between each other to construct the vegetation index combination for ginkgo leaf yield estimation. **Figure 6a** is the ranking of the importance of 7 vegetation index features. It can be seen from the graph that the ReCI vegetation index exhibited the highest importance of 0.3367, followed by the NDVI vegetation index, and the vegetation index with the smallest feature importance is GNDVI. **Figure 6b** depicts five main vegetation indices selected by random forest stepwise regression method, which are ReCI, NDVI, GNDVI, SAVI and MSAI.

**Figure 7:**
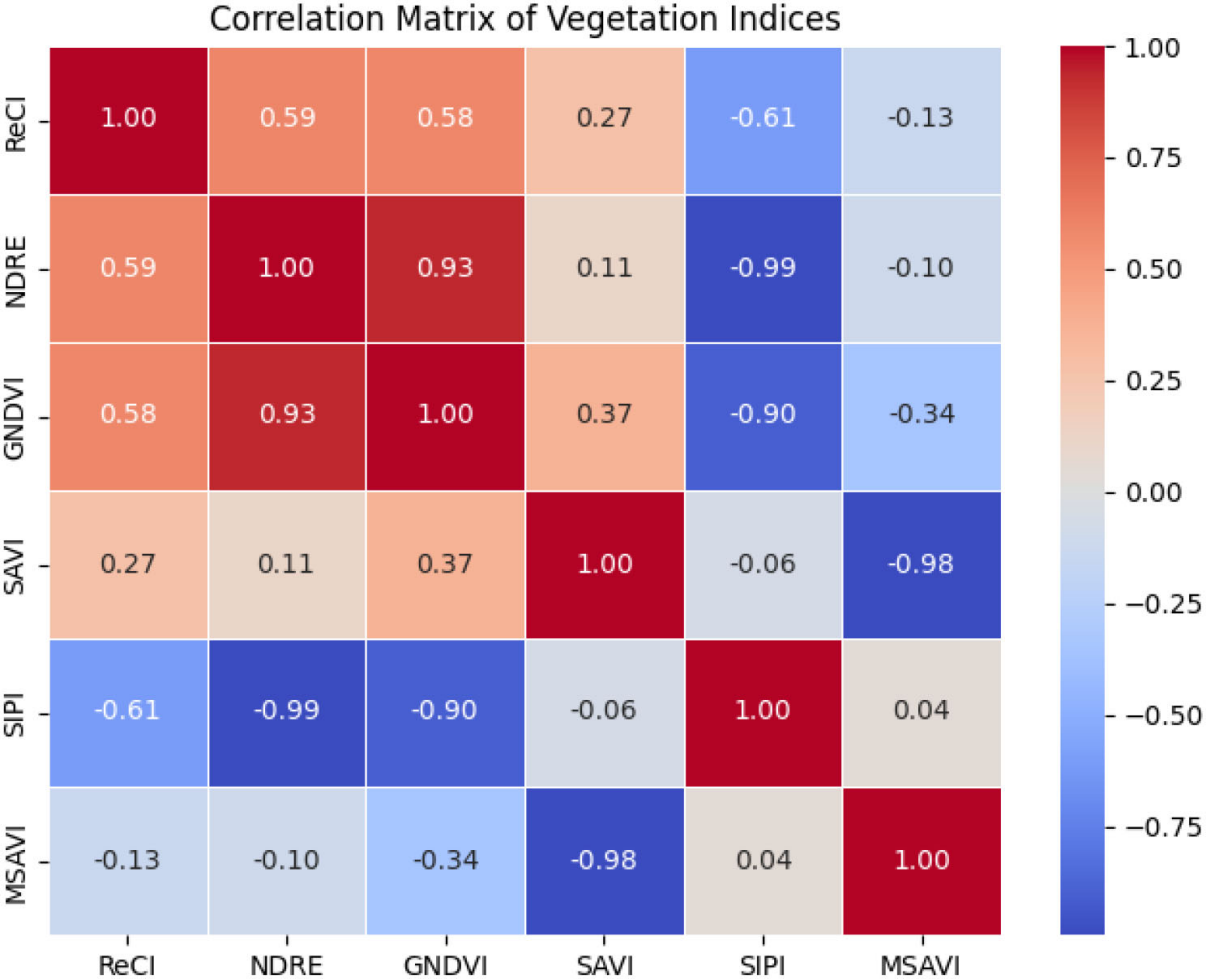
Autocorrelation analysis of vegetation index.

The correlation analysis results of **Figure 7** show that GNDVI and NDRE were highly positively correlated (r = 0.93), indicating that they have similar spectral response characteristics and can be used interchangeably to reduce data redundancy. However, GNDVI and SIPI (r = -0.90), SAVI and MSAVI (r = -0.98), and SIPI and NDRE (r = -0.99) showed significant negative correlations. These index combinations may represent different vegetation physiological characteristics or environmental stress responses. Therefore, SAVI, MSAVI, NDRE and SIPI were finally selected as the representative vegetation index combination, which can not only fully reflect the vegetation status, but also effectively avoid information redundancy.

Based on the above analysis, the spectral indices suitable for model input include SAVI, MSAVI, NDRE and SIPI. The combination of these indices has low redundancy information, which can effectively reflect the characteristic information of vegetation from multiple dimensions, and provides a more comprehensive and accurate input variable for the estimation of ginkgo leaf yield.

### 3.3 Feature band extraction

In this study, a variety of feature band screening methods were used to analyze the spectral data preprocessed by SNV. Among them PSO, SPA, PSAMA, LASSO and CARS were used to filter the characteristic bands (10,10,20,20 and 23, respectively), which were evenly distributed in the range of 400 ~ 1000 nm, effectively reducing redundant information and retaining the most representative spectral features. In contrast, the 21 characteristic bands screened by PCA were mainly concentrated in the short-wave region of 400 ~ 450 nm, showing unique wavelength selection characteristics. The results are shown in **Table 3**. Different feature selection methods have both commonalities in band distribution (most methods select wide spectral range features) and their own characteristics (PCA focusing on shortwave regions), which provides a variety of feature selection schemes for subsequent spectral analysis.

**Table 3:** Feature bands selected by different algorithms.

Algorithm of feature band screening	Filtered bands/nm	Number of bands
Particle Swarm Optimization (PSO)	408,438,446,535,685,691,701, 728,892,951	10
Particle Swarm Attention Mechanism Algorithm (PSAMA)	440,446,473,491,545,551,645,666,672,704,723, 734,766,800,884,925,930,962,970,989	20
Successive Projections Algorithm (SPA)	400,403,680,730,760,920,930,950,980,1000	10
Principal Component Analysis (PCA)	400,403,406,408,409,411,413,416,419,421,424,426,429,432,434,437,440,442,445,448,450	21
Least Absolute Shrinkage and Selection Operator (LASSO)	408,411,424,432,440,446,456,462,465,470,473,491,500,766,906,933,946,957,970,973	20
Competitive Adaptive Reweighted Sampling (CARS)	408,416,422,443,454,470,473,481,502,540,607,610,736,739,900,903,906,914,916,919,922,925,956	23

### 3.4 Prediction results using different machine learning and feature band screening methods

This study utilized full-band spectra, vegetation indices, and feature-selected spectral bands as input variables for a suite of machine learning and deep learning models. Specifically, A1 represents the complete set of 224 bands, while A2 to A8 correspond to features selected by vegetation indices, Particle Swarm Optimization (PSO), PSO with an attention mechanism, Successive Projections Algorithm (SPA), Principal Component Analysis (PCA), Least Absolute Shrinkage and Selection Operator (LASSO), and Competitive Adaptive Reweighted Sampling (CARS), respectively. To enhance estimation accuracy, A2 (spectral indices) and the Region of Interest Pixel set (denoted as B) were systematically combined with A1 and the bands from A3 to A8. Through systematic pairing with models including PLSR, RF, KNNR, LSTM, SVR, BiLSTM, and BiLSTM-GS, a total of 168 distinct data-model combinations were generated. All model hyperparameters were optimized via grid search cross-validation, with the final configurations established as follows: PLSR (n_components=10), RF (n_estimators=200), KNNR (n_neighbors=10), SVR (C = 10), LSTM (learning_rate=0.01), BiLSTM (learning_rate=0.01), and BiLSTM-GS (learning_rate=0.01, hidden_layer_sizes=50).

**Figure 8:**
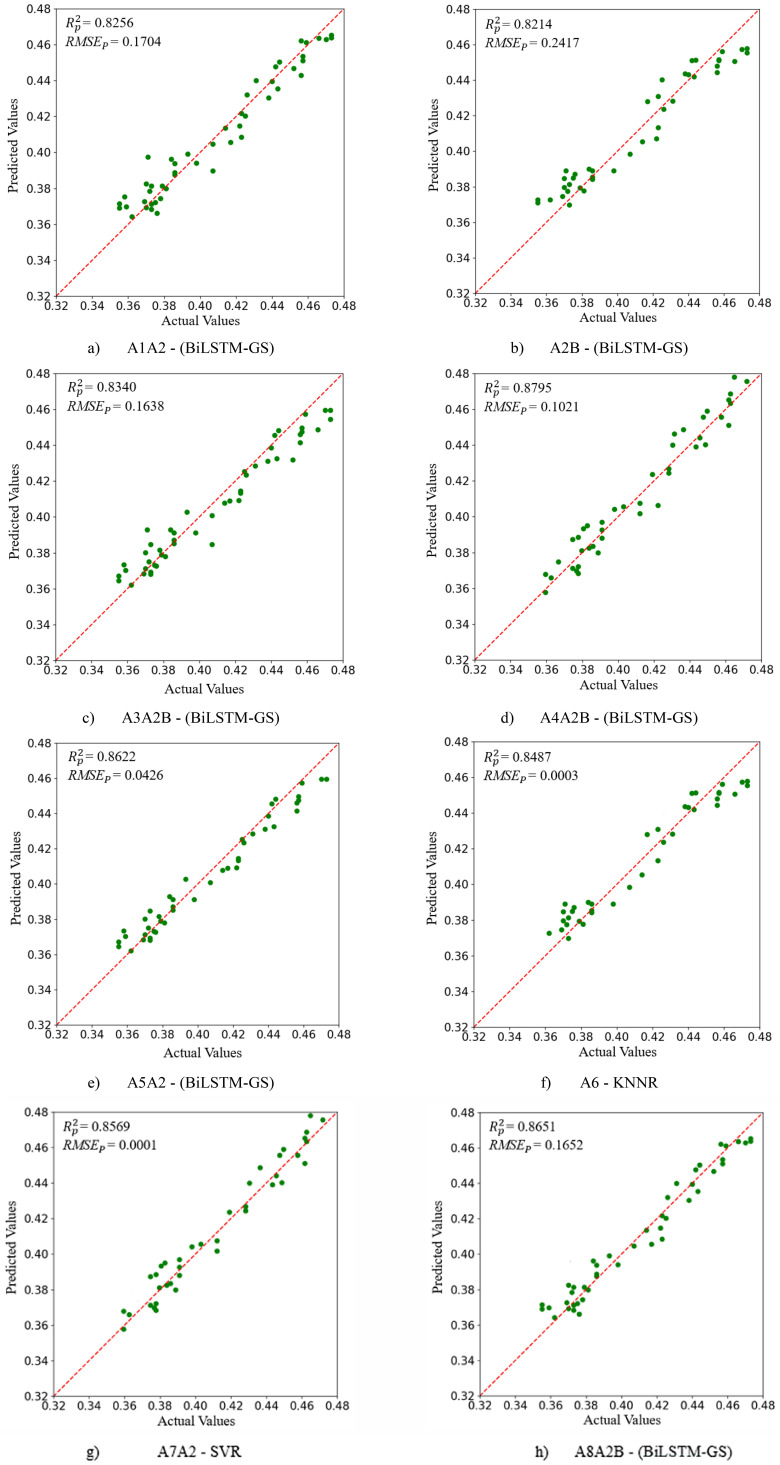
**(a–h)** represent the optimal yield prediction models corresponding to the combinations of the selected spectral bands (A1 to A8), the filtered vegetation indices, and the ROP (Region of Interest Pixels).

As shown in **Table 4**, when A4A2B was used as the input variable, the BiLSTM-GS model achieved the highest prediction accuracy ( $R_{P}^{2}\;$ = 0. 8795, $RMSE_{p}$ = 0.1021). To visually illustrate the optimal prediction yield models corresponding to the combination of A1 ~ A8 bands and spectral vegetation indices, eight regression diagrams were used, as shown in **Figure 8**.

**Table 4:** Ginkgo biloba leaf chlorophyll content prediction model results.

Input variable	Modeling method	$R_{C}^{2}$	$RMSE_{c}$	$R_{P}^{2}$	$RMSE_{p}$
A1	PLSR	0.9324	0.0221	0.7831	0.0318
	RF	0.9421	0.0181	0.7902	0.0221
	KNNR	0.8234	0.0003	0.6911	0.0005
	LSTM	0.2443	0.0012	0.1846	0.0011
	SVR	0.9669	0.0001	0.8053	0.0001
	BiLSTM	0.8824	0.0112	0.7621	0.0332
	(BiLSTM-GS)	0.9354	0.0181	0.7541	0.0423
A1B	PLSR	0.9256	0.0021	0.7654	0.0211
	RF	0.9624	0.0079	0.6652	0.0213
	KNNR	0.8121	0.0003	0.6832	0.0005
	LSTM	0.2425	0.0013	0.1845	0.0011
	SVR	0.9534	0.0001	0.7821	0.0001
	BiLSTM	0.9345	0.1071	0.7956	0.1456
	(BiLSTM-GS)	0.9324	0.1545	0.7733	0.1517
A1A2	PLSR	0.9347	0.0079	0.7315	0.0119
	RF	0.9417	0.0077	0.6012	0.2181
	KNNR	0.8323	0.0003	0.7012	0.0005
	LSTM	0.3356	0.0012	0.2956	0.0016
	SVR	0.9521	0.0001	0.6984	0.0001
	BiLSTM	0.9447	0.1145	0.7322	0.1458
	(BiLSTM-GS)	0.9347	0.1046	0.8256	0.1704
A1A2B	PLSR	0.9627	0.0078	0.7258	0.0124
	RF	0.9356	0.0076	0.6145	0.0256
	KNNR	0.8458	0.0003	0.7815	0.0005
	LSTM	0.3357	0.0012	0.2856	0.0016
	SVR	0.9625	0.0001	0.6856	0.0001
	BiLSTM	0.9432	0.1025	0.7232	0.0225
	(BiLSTM-GS)	0.9458	0.1033	0.7811	0.1625
A2	PLSR	0.9021	0.0201	0.4350	0.0282
	RF	0.9570	0.0080	0.6287	0.0231
	KNNR	0.8325	0.0003	0.7625	0.0005
	LSTM	0.3343	0.0012	0.2928	0.0016
	SVR	0.9322	0.0001	0.6654	0.0001
	BiLSTM	0.9174	0.0256	0.7200	0.3561
	(BiLSTM-GS)	0.9344	0.3838	0.7902	0.3579
A2B	PLSR	0.9334	0.0168	0.6678	0.0045
	RF	0.9541	0.0276	0.7215	0.0258
	KNNR	0.8856	0.0003	0.7659	0.0005
	LSTM	0.3459	0.0012	0.2859	0.0016
	SVR	0.9542	0.0001	0.6854	0.0001
	BiLSTM	0.9324	0.0217	0.7542	0.0215
	(BiLSTM-GS)	0.9657	0.025	0.8214	0.2417
A3	PLSR	0.8855	0.0140	0.7921	0.0212
	RF	0.9051	0.0278	0.6453	0.0576
	KNNR	0.8845	0.0003	0.7589	0.0005
	LSTM	0.3357	0.0012	0.2757	0.0016
	SVR	0.9534	0.0001	0.6751	0.0001
	BiLSTM	0.8845	0.0384	0.7014	0.0332
	(BiLSTM-GS)	0.9533	0.0257	0.7845	0.0323
A3B	PLSR	0.9425	0.0317	0.6954	0.0325
	RF	0.9514	0.0068	0.6957	0.0151
	KNNR	0.8824	0.0003	0.7589	0.0005
	LSTM	0.3468	0.0012	0.2958	0.0016
	SVR	0.9457	0.0001	0.6578	0.0001
	BiLSTM	0.9633	0.1122	0.7832	0.0189
	(BiLSTM-GS)	0.9422	0.0256	0.7789	0.1659
A3A2	PLSR	0.8327	0.0334	0.6241	0.0229
	RF	0.9499	0.0086	0.5064	0.0267
	KNNR	0.8853	0.0003	0.7128	0.0005
	LSTM	0.3365	0.0012	0.2845	0.0016
	SVR	0.9247	0.0001	0.6941	0.0001
	BiLSTM	0.9212	0.1165	0.7602	0.2146
	(BiLSTM-GS)	0.8656	0.1544	0.7659	0.2142
A3A2B	PLSR	0.8485	0.0225	0.5921	0.0265
	RF	0.9321	0.0078	0.6214	0.0325
	KNNR	0.8826	0.0003	0.7133	0.0005
	LSTM	0.3345	0.0012	0.2625	0.0016
	SVR	0.9326	0.0001	0.7556	0.0001
	BiLSTM	0.9215	0.1026	0.7626	0.2021
	(BiLSTM-GS)	0.8854	0.1577	0.8340	0.1638
A4	PLSR	0.8911	0.3154	0.7751	0.4273
	RF	0.9355	0.0102	0.6972	0.0206
	KNNR	0.8832	0.0003	0.7142	0.0005
	LSTM	0.3256	0.0012	0.2524	0.0016
	SVR	0.9235	0.0001	0.7332	0.0001
	BiLSTM	0.8933	0.0246	0.7712	0.0421
	(BiLSTM-GS)	0.9512	0.0117	0.7969	0.0235
A4B	PLSR	0.8824	0.0217	0.6325	0.0256
	RF	0.9145	0.0088	0.6954	0.0227
	KNNR	0.8825	0.0003	0.7132	0.0005
	LSTM	0.3326	0.0012	0.2845	0.0016
	SVR	0.9247	0.0001	0.7569	0.0001
	BiLSTM	0.9215	0.1031	0.8254	0.0332
	(BiLSTM-GS)	0.9256	0.0795	0.8341	0.1897
A4A2	PLSR	0.8541	0.0217	0.6874	0.0213
	RF	0.9321	0.0121	0.6789	0.0258
	KNNR	0.8844	0.0003	0.7245	0.0005
	LSTM	0.3318	0.0012	0.2832	0.0016
	SVR	0.9312	0.0001	0.7421	0.0001
	BiLSTM	0.9328	0.0545	0.7427	0.1032
	(BiLSTM-GS)	0.9425	0.0617	0.8021	0.0856
A4A2B	PLSR	0.8625	0.0218	0.6562	0.0217
	RF	0.9256	0.0129	0.6654	0.0235
	KNNR	0.8838	0.0003	0.7238	0.0005
	LSTM	0.3412	0.0012	0.2858	0.0016
	SVR	0.9314	0.0001	0.7325	0.0001
	BiLSTM	0.9456	0.0325	0.7328	0.0256
	(BiLSTM-GS)	0.9422	0.0817	0.8795	0.1021
	PLSR	0.9324	0.0221	0.7831	0.0318
	RF	0.9421	0.0181	0.7902	0.0221
A5	KNNR	0.8603	0.0002	0.7436	0.0004
	LSTM	0.3418	0.0012	0.2842	0.0016
	SVR	0.9315	0.0001	0.7412	0.0001
	BiLSTM	0.8824	0.0112	0.7621	0.0332
	(BiLSTM-GS)	0.9354	0.0181	0.7541	0.0423
A5B	PLSR	0.9324	0.0115	0.7624	0.0312
	RF	0.9425	0.0141	0.7617	0.0125
	KNNR	0.8633	0.0017	0.7512	0.0021
	LSTM	0.2754	0.0158	0.2944	0.0451
	SVR	0.9321	0.0001	0.7418	0.0001
	BiLSTM	0.9345	0.0121	0.7578	0.0158
	(BiLSTM-GS)	0.9358	0.0178	0.8208	0.0224
A5A2	PLSR	0.9524	0.0325	0.7415	0.0135
	RF	0.9316	0.0124	0.7352	0.0164
	KNNR	0.8066	0.0003	0.7021	0.0005
	LSTM	0.3021	0.0012	0.2854	0.0010
	SVR	0.9326	0.0001	0.7215	0.0001
	BiLSTM	0.9433	0.0154	0.7435	0.0158
	(BiLSTM-GS)	0.9447	0.0125	0.8622	0.0426
A5A2B	PLSR	0.9625	0.0244	0.7423	0.0323
	RF	0.9416	0.0132	0.7456	0.0118
	KNNR	0.8356	0.0003	0.6868	0.0005
	LSTM	0.3029	0.0012	0.2824	0.0010
	SVR	0.9521	0.0001	0.7524	0.0001
	BiLSTM	0.9332	0.0156	0.7318	0.0118
	(BiLSTM-GS)	0.9254	0.0135	0.8327	0.0565
A6	PLSR	0.9324	0.0221	0.7831	0.0318
	RF	0.9421	0.0181	0.7902	0.0221
	KNNR	0.9276	0.0001	0.8487	0.0003
	LSTM	0.0287	0.0016	0.0192	0.0014
	SVR	0.9524	0.0001	0.7621	0.0001
	BiLSTM	0.8824	0.0112	0.7621	0.0332
	(BiLSTM-GS)	0.9354	0.0181	0.7541	0.0423
A6B	PLSR	0.9526	0.0225	0.7354	0.0156
	RF	0.9421	0.0146	0.7541	0.0236
	KNNR	0.9468	0.0001	0.8145	0.0001
	LSTM	0.3716	0.0011	0.3059	0.0010
	SVR	0.9417	0.0001	0.7456	0.0001
	BiLSTM	0.8625	0.0113	0.7754	0.0364
	(BiLSTM-GS)	0.9521	0.0145	0.8215	0.0336
A6A2	PLSR	0.7547	0.0204	0.7457	0.0115
	RF	0.9538	0.0232	0.7459	0.0118
	KNNR	0.8094	0.0003	0.6486	0.0006
	LSTM	0.3720	0.0011	0.3089	0.0010
	SVR	0.9357	0.0001	0.7425	0.0001
	BiLSTM	0.8818	0.0154	0.7457	0.0112
	(BiLSTM-GS)	0.9627	0.0134	0.8151	0.0325
A6A2B	PLSR	0.7857	0.0145	0.7326	0.0135
	RF	0.9524	0.0136	0.7638	0.0115
	KNNR	0.8635	0.0003	0.6745	0.0006
	LSTM	0.3457	0.0011	0.3159	0.0010
	SVR	0.9356	0.0001	0.7021	0.0001
	BiLSTM	0.8847	0.0135	0.7326	0.0145
	(BiLSTM-GS)	0.9547	0.0121	0.8025	0.0184
A7	PLSR	0.9324	0.0221	0.7831	0.0318
	RF	0.9421	0.0181	0.7902	0.0221
	KNNR	0.8325	0.0002	0.7188	0.0005
	LSTM	0.3453	0.0011	0.3145	0.0010
	SVR	0.9478	0.0001	0.7126	0.0001
	BiLSTM	0.8824	0.0112	0.7621	0.0332
	(BiLSTM-GS)	0.9354	0.0181	0.7541	0.0423
A7B	PLSR	0.7625	0.0201	0.7357	0.0185
	RF	0.9524	0.0145	0.7625	0.0136
	KNNR	0.8725	0.0002	0.7012	0.0005
	LSTM	0.3852	0.0010	0.3025	0.0009
	SVR	0.9524	0.0001	0.7212	0.0001
	BiLSTM	0.8856	0.0124	0.7627	0.0103
	(BiLSTM-GS)	0.9457	0.0118	0.8225	0.0147
A7A2	PLSR	0.7644	0.0201	0.7325	0.0133
	RF	0.9623	0.0115	0.7221	0.0115
	KNNR	0.8316	0.0002	0.7223	0.0005
	LSTM	0.2904	0.0012	0.2535	0.0010
	SVR	0.9670	0.0001	0.8569	0.0001
	BiLSTM	0.8835	0.0118	0.7326	0.0108
	(BiLSTM-GS)	0.9533	0.0221	0.8225	0.0223
A7A2B	PLSR	0.7856	0.0214	0.7315	0.0115
	RF	0.9514	0.0124	0.7216	0.0135
	KNNR	0.8354	0.0002	0.7235	0.0005
	LSTM	0.2963	0.0012	0.2215	0.0011
	SVR	0.9554	0.0001	0.7956	0.0001
	BiLSTM	0.8824	0.0152	0.7456	0.0125
	(BiLSTM-GS)	0.9623	0.0203	0.8213	0.0263
A8	PLSR	0.7634	0.0202	0.7301	0.0103
	RF	0.9621	0.0135	0.7214	0.0124
	KNNR	0.8025	0.0003	0.6270	0.0006
	LSTM	-0.0046	0.0014	-0.0100	0.0017
	SVR	0.9622	0.0001	0.8297	0.0001
	BiLSTM	0.8812	0.0145	0.7432	0.0115
	(BiLSTM-GS)	0.9615	0.0201	0.8323	0.0154
A8B	PLSR	0.7425	0.0200	0.6524	0.0223
	RF	0.9515	0.0045	0.7545	0.0094
	KNNR	0.8195	0.0003	0.7054	0.0005
	LSTM	0.2952	0.0013	0.1854	0.0012
	SVR	0.9554	0.0001	0.7951	0.0001
	BiLSTM	0.8826	0.0115	0.7225	0.0125
	(BiLSTM-GS)	0.9626	0.0225	0.8245	0.0126
A8A2	PLSR	0.7123	0.0200	0.6824	0.0228
	RF	0.9635	0.0045	0.7621	0.0094
	KNNR	0.8157	0.0003	0.6910	0.0005
	LSTM	0.2441	0.0013	0.1847	0.0011
	SVR	0.9671	0.0001	0.8593	0.0001
	BiLSTM	0.8812	0.0123	0.7456	0.0128
	(BiLSTM-GS)	0.9565	0.0233	0.8245	0.0217
A8A2B	PLSR	0.7236	0.0196	0.6926	0.0216
	RF	0.9618	0.0043	0.7413	0.0078
	KNNR	0.8821	0.0003	0.6215	0.0006
	LSTM	0.3521	0.0013	0.2854	0.0011
	SVR	0.9514	0.0001	0.7852	0.0001
	BiLSTM	0.8884	0.1406	0.8241	0.1592
	(BiLSTM-GS)	0.9624	0.2198	0.8651	0.1652

**Figure 9:**
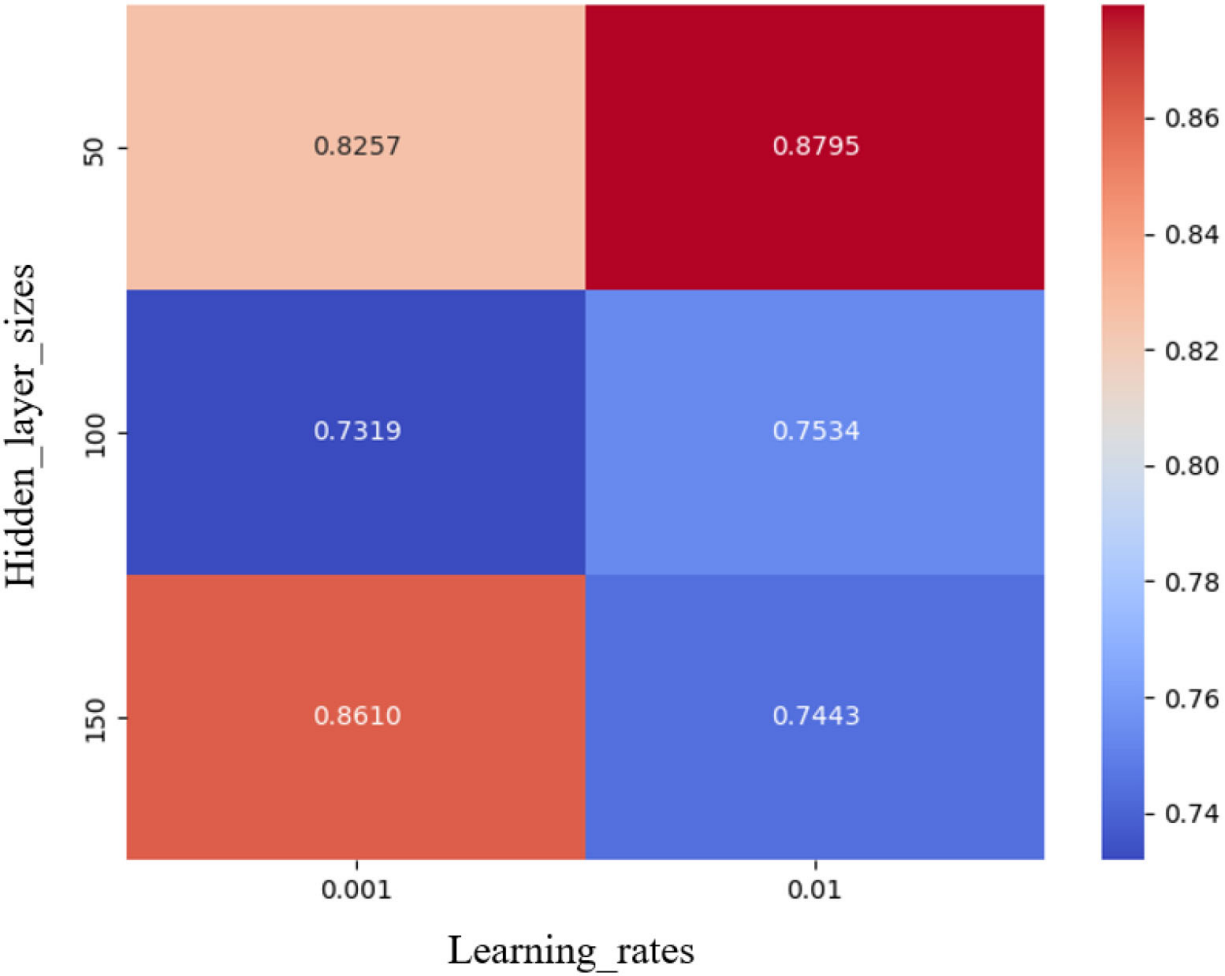
BILSTM hyperparameter optimization. The deeper the red color, the better the fitting effect and the better the prediction performance.

To obtain the best accuracy of the model (BiLSTM-GS), the grid SearchCV algorithm was used to optimize the hyper-parameters of the BiLSTM model, and the hyper-parameter learning rate and the size of the hidden layer was used as input. The (BiLSTM-GS) model determination coefficient ( $R_{P}^{2}$) is the output to draw the heat map. The results are shown in **Figure 9**. The deeper the red color, the higher the determination coefficient ( $R_{P}^{2}$) corresponding to the (BiLSTM-GS) model, and the better the fitting performance. When the (BiLSTM-GS) model obtained the best $R_{P}^{2}$ = 0. 8795, the corresponding optimal learning rate and hidden layer size were 0.01 and 50, respectively.

### 3.5 Model generalization ability validation

To validate the model’s generalization performance, an external public dataset, LOPEX1993, was employed. LOPEX1993 is an open spectral data set focusing on vegetation research, with rich spectral information of vegetation. In this study, PLSR, RF, KNNR, SVR, BiLSTM and BiLSTM-GS were used to detect the chemical component content of the data set. **Table 5** shows that the (BiLSTM-GS) model achieved the highest detection accuracy. Through the above comparative analysis, it can be found that BiLSTM has the following advantages over models such as PLSR, RF, KNNR and SVR. First, as the only model that can capture sequence context information in both directions, BiLSTM does not need to rely on manual design features (such as RF requires feature engineering, KNNR needs to define distance metrics). Second, its unique forgetting gate, input gate and output gate mechanism can dynamically adjust the information flow and effectively solve the long-term dependence problem (better than PLSR). In addition, in sequence data tasks such as natural language processing, BiLSTM performs significantly better than traditional methods (such as SVR). The hyperparameters optimized by Grid SearchCV further improve the performance of the model, so that the trained model can achieve higher prediction accuracy. Therefore, the prediction effect of the (BiLSTM-GS) model on the LOPEX1993 public dataset is significantly better than other comparison models.

**Table 5:** Lopex1993 vegetation data model test.

Modeling method	$R_{C}^{2}$	$RMSE_{c}$	$R_{P}^{2}$	$RMSE_{p}$
PLSR	0.9657	20.9183	0.8469	28.6118
RF	0.9883	12.2336	0.6527	26.9993
KNNR	0.9631	0.6779	0.7734	0.0585
SVR	0.7911	0.3566	0.6688	0.8384
BiLSTM	0.8824	0.0112	0.7621	0.0332
(BiLSTM-GS)	0.9259	0.0407	0.8503	0.0656

### 3.6 Discussion

This study systematically developed and validated a hyperspectral inversion model for ginkgo leaf yield prediction by integrating advanced preprocessing, feature selection, and machine learning techniques. The optimal SNV- (SAVI - MSAVI – NDRE - SIPI - ROP) - (BiLSTM-GS) model achieved superior performance ( $R_{P}^{2}$ = 0.8795), demonstrating the significant potential of airborne hyperspectral imaging for non-destructive yield assessment in economic forestry.

Our findings reveal that SNV preprocessing yielded optimal PLSR performance ( $R_{P}^{2}\;$ = 0.7831), aligning with regarding its robustness in mitigating scattering effects (Jin et al., 2023). The proposed PSAMA algorithm effectively identified 20 key bands across 400–1000 nm, demonstrating superior convergence and computational efficiency compared to traditional methods, consistent with recent trends in combining optimization algorithms with attention mechanisms (Lang et al., 2024). To effectively capture key yield-related physiological traits and minimize redundancy, we selected SAVI, MSAVI, NDRE, and SIPI as the optimal vegetation indices through random forest regression (Wang et al., 2024; Tooley et al., 2024).

The model’s superior performance stems from a strategic data fusion of feature bands, vegetation indices, and Ginkgo canopy region of interest pixel (ROP) sets, an approach consistent with established research (Ceriani et al., 2025). The BiLSTM-GS component particularly outperformed traditional methods due to its ability to capture bidirectional contextual dependencies in spectral sequences, a capability aligned with established mechanistic principles (Zhang et al., 2025). Comparative analysis shows our results exceed in apple yield prediction ( $R_{P}^{2}\;$=0.78) but trail in corn silage monitoring (>90%), highlighting the crop-specific nature of model performance (Burglewski et al., 2024).

While promising results have been achieved, this study has several limitations: limited generalizability due to single-site validation, the high computational demands of the BiLSTM-GS model, and the time-consuming manual ROI extraction. Future work should prioritize: 1) multi-site and multi-temporal validation to assess model robustness; 2) model lightweighting through compression techniques; 3) automated ROI extraction using deep learning; 4) multi-modal data fusion with LiDAR and meteorological data; 5) applying explainable AI methods to bridge the gap between model performance and biological insight; and 6) integrating various vegetation indices (e.g., TVI, MCARI, MTVI, NDI) to leverage their complementarity for enhancing the model’s characterization of multidimensional vegetation physio-biochemical traits.

In conclusion, while the proposed framework shows strong innovation and performance, addressing these limitations through integrated approaches will be crucial for its operational adoption in precision forestry and agriculture.

## 4 Conclusion

In this study, an innovative method based on hyperspectral imaging technology (400 ~ 1000 nm spectral range) was proposed to realize the non-destructive yield prediction of Ginkgo biloba leaves by integrating canopy spectral data and measured yield values. Through the systematic evaluation of five spectral preprocessing methods (MSC, SG, SNV, FD, SS), SNV was determined as the optimal preprocessing scheme and used as the basis for subsequent model training. PSO, PSAMA, SPA, PCA, LASSO and CARS algorithms were used for feature selection, and 10, 20, 10, 21, 20 and 23 characteristic wavelengths were extracted respectively. Additionally, the most representative SAVI, MSAVI, NDRE and SIPI vegetation indices were selected from the seven candidate indices by random forest regression analysis. The SNV- (SAVI - MSAVI – NDRE - SIPI - ROP) - (BiLSTM-GS) prediction model is constructed by innovatively fusing the Region of Interest Pixel (ROP) data, and the BiLSTM is optimized by Grid SearchCV (learning rate: 0.01, Hidden layers: 50). The model showed a prediction accuracy of $R_{c}^{2}$ = 0.9422 ( $RMSE_{c}\;$ = 0.0817) for the calibration set performance index and $R_{P}^{2}$ = 0.8795 ( $RMSE_{p}\;$ = 0.1021) for the prediction set result, thus establishing a robust technical framework for the non-destructive yield assessment of ginkgo planting.

## Data Availability

The raw data supporting the conclusions of this article will be made available by the authors, without undue reservation.

## References

[B1] Andrei G. Y. Costa E. M. Anjos L. H. C. D. Marcondes R. A.T. (2023). Enhancing soil mapping with hyperspectral subsurface images generated from soil lab vis-swir spectra tested in Southern Brazil. Geoderma Reg. 33, e00641–e00641. doi: 10.1016/j.geodrs.2023.e00641

[B2] Black D. Gill J. Xie A. Liquet B. Di leva A. Stummer W. . (2024). Deep learning-based hyperspectral image correction and unmixing for brain tumor surgery. iScience 27, 111273–111273. doi: 10.1016/j.isci.2024.111273, PMID: 39628576 PMC11613202

[B3] Bing L. Jun S. Ning Y. Xiao-hong W. Xin Z. (2018). Prediction of Tea Diseases Based on Fluorescence Transmission Spectrum and Texture of Hyperspectral Image. Spectroscopy and Spectral Analysis 39, 2515–2521. 10.3964/j.issn.1000-0593(2019)08-2515-07

[B4] Burglewski N. Srinivasagan S. Ketterings Q. Aardt V J. (2024). Spatial and spectral dependencies of maize yield estimation using remote sensing. Sensors 24, 3958–3958. doi: 10.3390/s24123958, PMID: 38931742 PMC11207318

[B5] Ceriani R. Brocco S. Monica P. Oggioni S. Vacchiano G. Motta R. . (2025). Hyperspectral and lidar space-borne data for assessing mountain forest volume and biomass. Int. J. Appl. Earth Obs Geoinf 141, 104614–104614. doi: 10.1016/j.jag.2025.104614

[B6] Chitra S. Chaudhry Muhammad M. A. Paliwal J. (2022). Classification of pulse flours using near-infrared hyperspectral imaging. LWT 154, 112799–112799. doi: 10.1016/j.lwt.2021.112799

[B7] Faehn C. Konert G. Keinänen M. Karppinen K. Krause K. (2024). Advancing hyperspectral imaging techniques for root systems: a new pipeline for macro- and microscale image acquisition and classification. Plant Methods 20, 171–171. 10.1186/s13007-024-01297-x, PMID: 39529150 PMC11555864

[B8] Ge Y. Atefi A. Zhang H. Chenyong Miao C. Raghuprakash Ramamurthy K. . (2019). High-throughput analysis of leaf physiological and chemical traits with vis–nir–swir spectroscopy: A case study with a maize diversity panel. Plant Methods 15, 1–12. doi: 10.1186/s13007-019-0450-8, PMID: 31391863 PMC6595573

[B9] Gila D. M. M. Martínez D. B. Satorres Martínez S. Marchal P. C. García J. G. (2024). Non-invasive detection of pesticide residues in freshly harvested olives using hyperspectral imaging technology. Smart Agric. Technol. 9, 100644–100644. doi: 10.1016/j.atech.2024.100644

[B10] Jin X. Han K. Zhao H. Wang Y. Chen Y. Yu J. L. (2023). Detection and coverage estimation of purple nutsedge in turf with image classification neural networks. Pest Manag Sci. 80, 2352–0094. doi: 10.1002/ps.8055, PMID: 38436512

[B11] Jong L. J. S. Post A. L. Geldof F. Dashtbozorg B. Ruers T. J. M. Sterenborg H. J. C. M. (2024). Separating Surface Reflectance from Volume Reflectance in Medical Hyperspectral Imaging. Diagnostics 14, 1812–1812. 10.3390/diagnostics14161812, PMID: 39202300 PMC11353750

[B12] Lang H. Bao W. Feng W. Qu K. Ma X. Zhang X. (2024). Hyperspectral and multispectral images fusion based on pyramid swin transformer. Infrared Phys. Technol. 143, 105617–105617. doi: 10.1109/IGARSS53475.2024.10642087

[B13] Li H. Liu P. Li Z. Xu C. Pan J. Zhou Y. . (2024). Valorization ofginkgo bilobaleaf powder as a substrate in king oyster mushroom ( Pleurotus eryngii ) cultivation. Life 14, 639–639. doi: 10.3390/life14050639, PMID: 38792659 PMC11123215

[B14] Li M. Yin H. Gu F. Duan Y. Zhuang W. Han K. . (2025). Recent advances and applications of nondestructive testing in aaricultural products: A review. Processes 13, 2674–2674. doi: 10.3390/pr13092674

[B15] Liang M. Wang Z. Lin Y Li C. Zhang L. Lin Y. (2024). Study on detection of pesticide residues in tobacco based on hyperspectral imaging technology. Front. Plant Sci. 15. doi: 10.3389/fpls.2024.1459886, PMID: 39403614 PMC11471546

[B16] Liu Y. Huawei X. Zhang Y. Che F. Shen N. Cui Y. (2022). Leaves, seeds and exocarp of ginkgo biloba L. (Ginkgoaceae): A comprehensive review of traditional uses, phytochemistry, pharmacology, resource utilization and toxicity. J. Ethnopharmacol 298, 115645–115645. doi: 10.1016/j.jep.2022.115645, PMID: 35988840

[B17] Long T. Tang X. Liang C. Wu B. Huang B. Lan Y. . (2024). Detecting bioactive compound contents in dancong tea using vnir-swir hyperspectral imaging and krr model with a refined feature wavelength method. Food Chem. 460, 140579–140579. doi: 10.1016/j.foodchem.2024.140579, PMID: 39126740

[B18] Lu F. Jiayi S. Zhang G. Nie F. Wang J. Dai X. (2024). Protective effect of ginkgobiloba (Ginkgoaceae family) leaf extracts on female drosophila melanogaster suffering from ultraviolet irradiation through activating the keap1-nrf2 signaling pathway. Biol. Bull. 51, 518–529. doi: 10.1007/s00299-013-1397-2, PMID: 23459862

[B19] Mao B. Cheng Q. Chen L Duan F. Sun X. Li Y. . (2024). Multi-random ensemble on partial least squares regression to predict wheat yield and its losses across water and nitrogen stress with hyperspectral remote sensing. Comput. Electron. Agric. 222, 109046–109046. doi: 10.1016/j.compag.2024.109046

[B20] Marchese C. Colella S. Brando V. E. Zoffoli M. L. Volpe G. (2024). Towards accurate L4 ocean colour products: interpolating remote sensing reflectance via dineof. Int. J. Appl. Earth Obs Geoinf 135, 104270–104270. doi: 10.1016/j.jag.2024.104270

[B21] Matinfar M. M. Mohammdi M. B. Karami M. (2025). Unmasking the brain in cocaine use disorder: A deep learning approach with graph convolutional networks and principal component analysis. Next Res. 2, 100304–100304. doi: 10.1016/j.nexres.2025.100304

[B22] Miettinen I. Zhang C. Alonso L. Marín B. F. Plazaola J. G. Grebe S. . (2024). Hyperspectral imaging reveals differential carotenoid and chlorophyll temporal dynamics and spatial patterns in scots pine under water stress. Plant Cell Environ. 48, 1535–1554. doi: 10.1111/pce.15225, PMID: 39462945 PMC11695750

[B23] Nikzad M. H. Rarani M. H. Rasti R. (2025). Long-short-term memory (Lstm)-based modeling of the stiffness of 3d-printed pla parts. Mater. Lett. 379, 137636–137636. doi: 10.1016/j.matlet.2024.137636

[B24] Qin A. Sun J. Zhu X. Li M. Li C. Wang L. . (2025). The yield estimation of apple trees based on the best combination of hyperspectral sensitive wavelengths algorithm. Sustainability 17, 518–518. doi: 10.3390/su17020518

[B25] Ren C. Xu Q. Meng Z. Pan J. S. (2024). Surrogate-assisted fully-informed particle swarm optimization for high-dimensional expensive optimization. Appl. Soft Comput. 167, 112464–112464. doi: 10.1016/j.asoc.2024.112464

[B26] Satyabrata D. Sujata C. Giri N. C. Agyekum E. B. AboRas K. M. (2024). Minimum noise fraction and long short-term memory model for hyperspectral imaging. Int. J. Comput. Int. Sys 17, 1–22. doi: 10.1007/s44196-023-00370-y

[B27] Sen D. Jain S. K. Rathee S. Umesh Patil K. U.K. Pandey V. (2025). Comprehensive insights into pathophysiology of alzheimer's disease: herbal approaches for mitigating neurodegeneration. Curr. Alzheimer Res. 21, 625–648. doi: 10.2174/0115672050309057240404075003, PMID: 38623983

[B28] Settembre G. Taggio N. Buono N. D. Esposito F. Lauro P. Aiello A. (2025). A land cover change framework analyzing wildfire-affected areas in bitemporal prisma hyperspectral images. Math. Comput. Simul 229, 855–866. doi: 10.1016/j.matcom.2024.10.034

[B29] Shezhou L. Cheng W. Xi X. Pan F. Qian M. Peng D. (2015). Retrieving aboveground biomass of wetland phragmites australis (Common reed) using a combination of airborne discrete-return lidar and hyperspectral data. Int. J. Appl. Earth Obs Geoinf 58, 107–117. doi: 10.1016/j.jag.2017.01.016

[B30] Shi Y. Wang F. Xie H. Fan B. Li L. Kong Z. . (2024). Model for prediction of pesticide residues in soybean oil using partial least squares regression with molecular descriptors selected by a competitive adaptive reweighted sampling algorithm. Agric. Commun. 2, 100053–100053. doi: 10.1016/j.agrcom.2024.100053

[B31] Song H. Mehdi S. R. Wan Q. Li Z. Li M. Wang M. . (2025). Compact staring-type underwater spectral imaging system utilizing K-nearest neighbor-based interpolation for spectral reconstruction. Opt Laser Technol. 181, 111880–111880. doi: 10.1016/j.optlastec.2024.111880

[B32] Souza L. L. D. Candeias D. N. C. Moreira E. D. T. Diniz P. H.G.D. Springer V. H. Fernandes D. D. S. . (2025). Uv–vis spectralprint-based discrimination and quantification of sugar syrup adulteration in honey using the successive projections algorithm (Spa) for variable selection. Chemometr Intell. Lab. 257, 105314–105314. doi: 10.1016/j.chemolab.2024.105314

[B33] Srivastava P. K. Gupta M. Singh U. Prasad R. Pandey P. C. Raghubanshi A. S. . (2020). Sensitivity analysis of artificial neural network for chlorophyll prediction using hyperspectral data. Environ. Dev. Sustain 23, 1–16. doi: 10.1007/s10668-020-00827-6

[B34] Stevens C. L. Adriaans G. M. C. Spooren C. E. G. M. Peters V. Pierik M. J. Weersma R. K. . (2025). Exploring diet categorizations and their influence on flare prediction in inflammatory bowel disease, using the sparse grouped least absolute shrinkage and selection operator method. Clin. Nutr. 47, 212–226. doi: 10.1016/j.clnu.2025.02.027, PMID: 40048994

[B35] Sun M. Yang Y. Li S. Yin D. Zhong G. Cao L. (2024). A study on hyperspectral soil total nitrogen inversion using a hybrid deep learning model cbiresnet-bilstm. Chem. Biol. Technol. Agric. 11, 157–157. doi: 10.1186/s40538-024-00681-y

[B36] Sun X. Zhang B. Dai M. Jing C. Ma K. Tang B. . (2024). Accurate irrigation decision-making of winter wheat at the filling stage based on uav hyperspectral inversion of leaf water content. Agr Water Manag 306, 109171–109171. doi: 10.1016/j.agwat.2024.109171

[B37] Tian J. Xu S. Wu Y. Shi Y. Duan Y. Li Z. . (2025). Authenticating vintage in white tea: appearance-taste-aroma-based three-in-one non-invasive anticipation. Food Res. Int. 199, 115394–115394. doi: 10.1016/j.foodres.2024.115394, PMID: 39658181

[B38] Tooley E.G. Nippert J. B. Ratajczak Z. (2024). Evaluating methods for measuring the leaf area index of encroaching shrubs in grasslands: from leaves to optical methods, 3-D scanning, and airborne observation. Agr For. Meteorol 349, 109964–109964. doi: 10.1016/j.agrformet.2024.109964

[B39] Tougas G. Wallis C. I.B. Lalibert E. Vellend M. (2025). Hyperspectral imaging has a limited ability to remotely sense the onset of beech bark disease. Remote Sens. Ecol. Conserv. 64, 199–209. doi: 10.1002/rse2.70013

[B40] Vitor D. A. C. Soliani A. G. Cerutti S. M. (2023). Standardized extract of ginkgo biloba treatment and novelty on the weak encoding of spatial recognition memory in rats. Learn. Mem 30, 85–95. doi: 10.1101/lm.053755.123, PMID: 37072140 PMC10165992

[B41] Wan S. Yang H. Lin J. Li J. Wang Y. Chen X. (2024). Improved whale optimization algorithm towards precise state-of-charge estimation of lithium-ion batteries via optimizing lstm. Energy 310, 133185–133185. doi: 10.1016/j.energy.2024.133185

[B42] Wang J. Chen C. Wang J. Yao Z. Wang Y. Zhao Y. . (2024). Ndvi estimation throughout the whole growth period of multi-crops using rgb images and deep learning. Agron 15, 63–63. doi: 10.3390/agronomy15010063

[B43] Wang X. Han J. Liu C. Feng T. (2024). Non-destructive assessment of apple internal quality using rotational hyperspectral imaging. Front. Plant Sci. 15. doi: 10.3389/fpls.2024.1432120, PMID: 39568455 PMC11577084

[B44] Wang J Chen C Wang J . (2024). Ndvi estimation throughout the whole growth period of multi-crops using rgb images and deep learning. Agron 15, 63–63. doi: 10.3390/agronomy15010063

[B45] Wei Y. Hu H. Xu H. Mao X. (2024). Identification of chrysanthemum variety via hyperspectral imaging and wavelength selection based on multitask particle swarm optimization. Spectrochim Acta A 322, 124812–124812. doi: 10.1016/j.saa.2024.124812, PMID: 39047665

[B46] Xu P. Fu L. Pan Y. Chen D. Yang S. Yang R. (2024). Identification of maize seed vigor based on hyperspectral imaging and deep learning. Bulletin of the National Research Centre 48, 84–84. 10.1186/s42269-024-01239-6

[B47] Xueyu T. Yanjie L. Yan W. Wang M. Tan Z. Jiang J. . (2021). Heritable variation in tree growth and needle vegetation indices of slash pine (Pinus elliottii) using unmanned aerial vehicles (Uavs). Ind. Crops Prod 173, 114073–114083. doi: 10.1016/j.indcrop.2021.114073

[B48] Xunlan L. Fangfang P. Zhaoxin W. Guohui H. Jianfei L. (2023). Non-destructive detection of protein content in mulberry leaves by using hyperspectral imaging. Frontiers in Plant Science 14, 1275004–1275004. 10.3389/fpls.2023.1275004, PMID: 37900759 PMC10602742

[B49] Yan K. Song X. Yang J. Xiao J. Xu X. Guo J. . (2025). Citrus huanglongbing detection: A hyperspectral data-driven model integrating feature band selection with machine learning algorithms. Crop Prot 188, 107008–107008. doi: 10.1016/j.cropro.2024.107008

[B50] Yue J. Wang J. Zhang Z. Li C. Yang H. Feng H. . (2024). Estimating crop leaf area index and chlorophyll content using a deep learning-based hyperspectral analysis method. Comput. Electron. Agric. 227, 109653–109653. doi: 10.1016/j.compag.2024.109653

[B51] Zhang S. Chen B. Chen C. Hovorka M. Qi J. Hu J. . (2025). Myoelectric signal and machine learning computing in gait pattern recognition for flat fall prediction. Med. Novel Technol. Devices 25, 100341–100341. doi: 10.1016/j.medntd.2024.100341

